# Table tennis playing styles require specific energy systems demands

**DOI:** 10.1371/journal.pone.0199985

**Published:** 2018-07-18

**Authors:** Fabio Milioni, Jorge Vieira de Mello Leite, Ralph Beneke, Rodrigo Araújo Bonetti de Poli, Marcelo Papoti, Alessandro Moura Zagatto

**Affiliations:** 1 Sao Paulo State University—UNESP, School of Sciences, Department of Physical Education, Post-Graduate Program in Movement Sciences, Laboratory of Physiology and Sport Performance (LAFIDE), Bauru, São Paulo, Brazil; 2 Federal University of Mato Grosso do Sul (UFMS), Post-Graduate Program in Health and Development in the Midwest Region, Campo Grande, Mato Grosso do Sul, Brazil; 3 Philipps-University Marburg, Marburg, Germany; 4 University of Sao Paulo, School of Physical Education and Sports of Ribeirao Preto, Ribeirao Preto, Sao Paulo, Brazil; Norwegian University of Science and Technology, NORWAY

## Abstract

The aim of the present study was to investigate the differences in energy system contributions and temporal variables between offensive and all-round playing styles. Fifteen male table tennis players (Offensive players: N = 7; All-round players: N = 8) participated in the study. Matches were monitored by a portable gas analyzer and the blood lactate responses was also measured. The contributions of the oxidative (W_OXID_), phosphagen (W_PCr_), and glycolytic (W_[La]_) energy systems were assumed as the oxygen consumption measured during the matches above of baseline value, the fast component of excess post-exercise oxygen consumption (EPOC_FAST_) measured after the matches, and the net of blood lactate concentration (Δ[La]), respectively. Energy systems contributions were not significantly different between the offensive and all-round playing styles (W_OXID_: 96.1±2.0 and 97.0±0.6%, *P* = 0.86; W_PCr_: 2.7±1.7 and 2.0±0.6%, *P* = 0.13; W_[La]_: 1.2±0.5 and 1.0±0.7%, *P* = 0.95; respectively), however, magnitude-based analysis of W_PCr_ presented *Likely higher* contribution for offensive compared to all-round players. Regarding temporal variables, only rate of shots presented higher values for offensive when compared to all-round players (*P* = 0.03), while the magnitude-based analysis presented *Very likely lower*, *Likely lower* and *Likely higher* outcomes of rate of shots, W_PCr_ and maximal oxygen consumption, respectively, for all-round players. Strong negative correlation was verified for offensive players between number of shots and W_PCr_ (r = -0.86, *P* = 0.01), while all-round players showed strong correlations between rally duration, W_OXID_ (r = 0.76, *P* = 0.03) and maximal oxygen consumption (r = 0.81, *P* = 0.03). Therefore, despite no differences in energy system contributions for offensive and all-round players, different playing styles seems to requires specific energy systems demands.

## Introduction

Table tennis is a racket sport practiced by millions of athletes and has been part of the Olympic program since 1988 [1]. Nevertheless, data on the metabolic profile of table tennis are limited to blood lactate concentration ([La]) and heart rate (HR) in official [2,3] and simulated matches [4], while respiratory measures have only been analyzed in simulated matches [5]. The detailed determination of the metabolic profiles of table tennis may help the development of sport specific training programs [5].

During table tennis matches the average values of [La], HR and oxygen consumption ($\dot{V}O_{2}$) are 2.0 mmol·L^−1^, 125 ± 22 bpm and 25.6 ± 10.1 mL·kg^-1^·min^-1^, respectively [3], characterizing the low physiological demand of the modality. Still, Zagatto et al. [3] reported an average rally duration of 3.4 ± 1.7 s, rest times between rallies corresponding to 8.1 ± 5.1 s, frequency of shots of 35.3 ± 7.7 balls·min^-1^ and a total match duration of 16.1±5.6 min, suggesting that aerobic energy metabolism is predominate when assumed the whole match with minor supplementation by high energy phosphates and, to a minor extent, glycolytic energy during short bouts of highly intense activity phases [3,4]. Recently, Zagatto et al. [5] provided strong evidence for the after mentioned assumptions based on the measurement of contribution of each metabolic pathway, corresponding to 96.5% (834.6 ± 242.2 [671.8–997.3] kJ) oxidative, 2.5% (19.3 ± 4.7 [16.2–22.4] kJ) high energy phosphates, and 1.0% (8.4 ± 6.4 [4.0–12.7] kJ) glycolytic energy, respectively. Oxidative and glycolytic energy demands were highly related to rally duration, while the rate of shots during rallies, which may serve as an index of exercise intensity, was significantly correlated with the high energy phosphates pathway and anaerobic capacity estimated by maximal accumulated oxygen deficit [5].

Table tennis has a wide variety of playing styles, comprising offensive, defensive and all-round, amongst other variations according match strategies [2]. These variations result in specific spatio-temporal activity patterns in terms of rally duration, rate of shots, and ball handling techniques and also in the use of specific equipment such as tacky or anti-spin rubbers, short and long pimples, wood and carbon blades–all factors likely to alter the activity pattern and thus specific metabolic profile of the sport [2]. Therefore, provide information regarding to energetic demand and activity pattern according to playing style may help for planning more efficient and specific training programs.

Thus, the purpose of this study was to analyze the potential effect of offensive and all-round playing styles on the specific metabolic profile and temporal variables of table tennis. We hypothesized that in comparison to all-round players, the offensive players would demonstrate more aggressive play determined by a higher rate of explosive actions during rallies, resulting in higher physiological demand, especially for the high-energy phosphate system.

## Methods

### Participants

Fifteen Brazilian national level male table tennis players (21 ± 4 years; 72.87 ± 15.6 kg; 174.9 ± 7.4 cm) who had been practicing 2–3 hours on 5 to 6 days per week for at least 5 years (9.2±3.5 years) participated in the study. They were familiarized with all experimental procedures and equipment, and were instructed to maintain the same individual light meal at least 2 hours before each testing session. Furthermore, they were instructed to maintain hydration habits, not to perform additional physical activities, and to avoid alcohol or caffeine ingestion during the experimental period. The athletes were informed about experimental procedures and risks, and signed an informed consent form prior to participation in the study. All procedures were approved by the University’s Institutional Review Board for Human Subjects (Human Research Ethics Committee–CAAE: 10499512.0.0000.0021/2012) and were conducted in accordance with the Declaration of Helsinki.

### Experimental design

The athletes underwent three experimental sessions carried out at the same time of the day. Time intervals between tests were at least 48 h. On the first day, athletes performed a graded exercise test (GXT) to determine the maximal oxygen consumption ($\dot{V}O_{2\max}$) on running treadmill. In the second and third sessions, simulated table tennis matches were performed, one with and one without metabolic measurements (i.e., portable gas analysis system and blood lactate measurements).

### Physiological measures

During the GXT and simulated matches, $\dot{V}O_{2}$, carbon dioxide production ($\dot{V}{\mathrm{CO}}_{2}$) and pulmonary ventilation (${\dot{V}}_{E}$) were continuously monitored, breath-by-breath, by a portable gas analysis system (K4b^2^, Cosmed, Rome, Italy). The HR was also recorded by the portable gas analysis system (K4b^2^, Cosmed, Rome, Italy) combined with a heart rate monitor (T31, Polar Electro, Kempele, Finland). Additionally, in the simulated matches, the baseline of $\dot{V}O_{2}$ was monitored during 10 min of seated rest ($\dot{V}O_{2\mathrm{REST}}$), as well as the 5-min warm-up and after the match end (~7 min) for measuring the fast component of excess post-exercise oxygen consumption (EPOC_FAST_). The gas analyzer was calibrated before each session according to the manufacturer's instructions, using ambient air and sample gases with known composition (16.02% O_2_ and 5.06% CO_2_, White Martins, Osasco, Brazil). The flowmeter was calibrated with a 3-liter syringe (Hans Rudolph, model 5530, Kansas City, Missouri, USA). To decrease the discrepant values, all data obtained for the cardiorespiratory variables ($\dot{V}O_{2},\;\dot{V}{\mathrm{CO}}_{2},\;{\dot{V}}_{E}$ and HR) were smoothed using moving 5-second averages and interpolated to provide one value per second (OriginPro 8.0, Origin Lab Corporation, Microcal, MA, USA).

To determine the [La], blood samples (25 μL) were collected from the earlobe in the rest period before the match, immediately after the end of each set, and in the 3^rd^, 5^th^, and 7^th^ minute after the match and GXT. The blood samples were stored at -20ºC in tubes containing 50 μL of sodium fluoride (1%) and analyzed in an electrochemical lactate analyzer (Yellow Springs Instruments model 2300 Stat, Ohio, USA) (measurement error of +2%).

### Graded exercise test (GXT)

The GXT was performed on a motorized treadmill (ATL, Inbramed, Inbrasport, Porto Alegre, RS, Brazil) with a fixed treadmill incline of 1%. Prior to the test, the athletes performed a 5 min warm-up at 6.0 km·h^-1^. Five minutes after the warm-up, the GXT started at 9 km·h^−1^ with stage increments of 1.0 km·h^−1^ every 2 minutes until voluntary exhaustion. The highest $\dot{V}O_{2}$ average (i.e., $\dot{V}O_{2}$ average measured during the final 30 s of each stage) was defined as $\dot{V}O_{2\max}$ if at least two of the following criteria were achieved: the plateau in $\dot{V}O_{2}$ (variation in $\dot{V}O_{2}$ < 2.1 mL·kg^−1^·min^−1^ during the last two stages of exercise); maximal HR (HR_MAX_) ≥ 90% of predicted HR_MAX_ (220—age); respiratory exchange ratio (RER) ≥ 1.10 and peak [La] ≥ 8.0 mmol·L^−1^ [6]. If less than two of the criteria were achieved then a new test was performed.

### Simulated table tennis matches

The simulated table tennis matches were performed following the current rules of the International Table Tennis Federation, (i.e., playing area size, referee, scoreboard, rest time, between 4 to 7 sets with 4 sets won required to win the match). Match parings were random. Each athlete played two matches separated by a minimum interval of 48 hours. Physiological responses of each participant were monitored in one match only.

Despite subjective, the playing style (i.e., offensive or all-round) of each athlete was defined based on athletes and coaches agreement. Basically, offensive players define their tactical and technical aspects trying to make a winner (i.e., a rally-ending shot after which the ball bounces into the opponent’s side of the table and is not hit or touched by the opponent [1]), while all-round players usually return balls using less aggressive strokes and trying to force an error of the opponent [1]. Both, athlete and coach were inquired separately about how they defined the athlete playing style. If the answers (i.e., athlete and coach) agreed, the reported playing style was assumed. If not, a second coach should answer the same inquire about the athlete playing style. In all cases the athletes and coaches’ answers had agreed.

The $\dot{V}O_{2\mathrm{REST}}$ was obtained by the average of the last 30 s of $\dot{V}O_{2}$ measured during 10 min seated. The peaks $\dot{V}O_{2}\;(\dot{V}O_{2\mathrm{PEAK}})$ and HR (HR_PEAK_) of the match were determined as the average of five values around the highest $\dot{V}O_{2}$ and HR values, respectively. The determination of the means of $\dot{V}O_{2}\;(\dot{V}O_{2\mathrm{MEAN}})$ and HR (HR_MEAN_) of the match considered only the values obtained during sets (i.e., discarding the rest periods between sets). The [La] value at rest was obtained in the final minute of the rest period, while the mean of [La] of the match ([La]_MEAN_) was determined using the lactate values obtained after each set and the peak lactate ([La]_PEAK_) corresponded to the highest value obtained during the match.

All matches were video recorded (Sony Handycam DCR-SR47, Tokyo, Japan) for subsequent analysis of the temporal variables; rally duration, rest time between rallies, work-to-rest ratio, number of shots per rally, and rate of shots per rally, using reliable and reproducible procedures adopted in a previous study (ICC = 0.78, *P* < 0.001 for rally duration and ICC = 0.95, *P* < 0.001 for the rest time between rallies) [3].

### Energy system contributions measured during simulated table tennis matches

The net oxidative energy (W_OXID_) was estimated by subtracting the $\dot{V}O_{2\mathrm{REST}}$ value multiplied by match time from the integrated $\dot{V}O_{2}$ area during the entire match including the breaks between sets [5,7,8]. The phosphagen system contribution (W_PCr_) was considered to be the EPOC_FAST_ [7], estimated by multiplication of the amplitude (A_1_) and the time constant (*τ*_1_) (Eq 1) fitted by a biexponential model using (OriginPro 8.0, Origin Lab Corporation, Microcal, MA, USA) (Eq 2) [5,8–10]. The $\dot{V}O_{2}$ data in the breaks between each rally and between sets were not considered as W_PCr_ contribution due to the very short recovery time (i.e., ~8-s) [3] and because the athletes often performed some low intensity activities during these breaks, such as jogging to catch the ball, using the towel or keeping the body warm (prepared), which maintained the increase in $\dot{V}O_{2}$ and could result in overestimation of the W_PCr_ [11]. Thus, the $\dot{V}O_{2}$ measured in all breaks was considered only as aerobic energy. Finally, the glycolytic system contribution (W_[La]_) was estimated by subtracting rest [La] from post-exercise lactate (Δ[La]), considering 1 mmol·L^-1^of the Δ[La] to be equivalent to 3 mL O_2·_kg^-1^ body mass [12]. The W_[La]_ was calculated for each set (blood lactate after the set minus the resting value) and the W_[La]_ of the total match corresponded to the sum of all Δ[La] values.

$$W_{\mathrm{PCr}}=A_{1}*\tau_{1}$$ (1)

$$\dot{V}O_{2(t)}=\dot{V}O_{2\mathrm{REST}}+A_{1}*[e^{-(t-\delta )/\tau 1}]+A_{2}*[e^{-(t-\delta )/\tau 2}]$$ (2)

where $\dot{V}O_{2(t)}$ is oxygen consumption at time *t*, $\dot{V}O_{2\mathrm{REST}}$ is baseline oxygen consumption, A is amplitude, *δ* is the time delay, *τ* is a time constant, and 1 and 2 denote the fast and slow components, respectively.

An energy equivalent related to the work done by each energy system of 20.9 kJ per L·O_2_^-1^ was considered as used previously in rowing [13], rock climbing [14], table tennis [5,15], treadmill running [16], repeated sprints [8], and others. The total amount of anaerobic work (W_ANAER_) corresponded to the sum of W_[La]_ and W_PCr_ and the total amount of work corresponded to the sum of W_OXID_, W_[La]_ and W_PCr_ (W_TOTAL_). The energy system contributions were analyzed for all players (n = 15) and also for playing style (offensive style players vs all-round style players).

### Statistical analysis

The results are presented as mean ± standard deviation and 95% confidence interval (95%CI). Initially, the data were analyzed by the Shapiro Wilk test to assess data normality, which allowed parametric analysis. To equalize and compare energy contributions, physiological responses, and temporal variables, only the first three sets and the final set played in the matches were selected, considering that only a few matches contained 5^th^, 6^th^, and 7^th^ sets [5].

The unpaired *t* test was used to compare the energy system contributions between offensive and all-round playing style. The Pearson’s product-moment correlation test was used to evaluate the possible associations between energy system contributions, and temporal variables for each group (offensive and all-round players). For all statistical analyses the software SPSS 17.0 was used and the significance level was set at *P* < 0.05.

Additionally, the magnitude-based analyses (Parallel group trials spreadsheet - http://www.sportsci.org/) was applied for comparison of offensive and all-round players. The pooled standard deviations of the moments of interest were multiplied by the smallest worthwhile change (SWC = 0.2) and assumed as Cohen’s d with the confidence interval pre-determined as 90% [17,18]. The threshold values of effect size (ES) for Cohen’s d statistics were present as module number and were classified as > 0.2 (small), > 0.5 (moderate), and > 0.8 (large). The quantitative chances of higher performance of offensive players, trivial or higher performance of all-round players were assessed qualitatively as follows: ≤ 1% most unlikely, > 1–5% very unlikely, > 5–25% unlikely, > 25–75% possibly, > 75–95% likely, > 95–99 very likely, > 99% most likely. If the chance of having higher performances for offensive and all-round were both > 5%, the true difference was assessed as unclear [17].

## Results

### Overall

A total of 15 (one for each athlete) matches were analyzed. There were no differences between groups for age (offensive: 21 ± 3 years; all-round: 20 ± 4 years–*P* = 0.33) and weight (offensive: 70.3 ± 5.7 kg; all-round: 74.9 ± 12.8 kg–*P* = 0.38). The scoring rule of 4 wins out of seven sets resulted in 5 ± 1 sets on average. This comprised six 4-set matches, three 5-set matches, five 6-set matches, and one 7-set matches. The mean duration of the matches was 23.6 ± 6.6 min. The effective playing time was 25.6 ± 2.5%. The overall of energy system contribution, temporal and physiological variables are present in Table 1 (Table 1).

**Table 1:** Comparisons between offensive and all-round playing style. Mean ± SD (95%CI).

		Overall	Offensive	All-round	*P*	(1-β)
***Energy systems contribution***						
**W**_**OXID**_	kJ %	778.2 ± 189.4 (682.3–874.0) 96.6 ± 1.4 (95.9–97.3)	768.3 ± 171.1 (687.0–849.7) 96.1 ± 2.2 (94.6–97.7)	786.8 ± 227.2 (678.8–894.8) 97.0 ± 0.6 (96.5–97.4)	0.86 0.34	86% 52%
**W**_**PCr**_	kJ %	17.2 ± 4.7 (14.8–19.5) 2.3 ± 1.2 (1.7–2.9)	19.4 ± 6.0 (16.5–22.2)2.7 ± 1.7 (1.4–4.0)	15.2 ± 2.9 (13.9–16.6) 2.0 ± 0.6 (1.6–2.4)	0.13 0.35	49% 52%
**W**_**[La]**_	kJ %	9.3 ± 6.0 (6.3–12.4) 1.1 ± 0.6 (0.8–1.4)	9.2 ± 4.5 (7.1–11.3) 1.2 ± 0.5 (0.8–1.6)	9.4 ± 7.8 (5.7–13.1) 1.0 ± 0.7 (0.5–1.5)	0.95 0.68	95% 70%
**W**_**ANER**_	kJ %	26.5 ± 8.2 (22.3–30.6) 3.4 ± 1.4 (2.7–4.1)	28.6 ± 8.1 (24.7–32.4) 3.9 ± 2.0 (2.3–5.4)	24.6 ± 8.9 (20.4–28.9) 3.0 ± 0.6 (2.6–3.5)	0.39 0.68	54% 52%
**W**_**TOTAL**_	kJ %	803.2 ± 192.3 (705.9–900.5) 100	796.9 ± 169.5 (716.3–877.5) 100	808.8 ± 233.5 (697.8–919.8) 100	0.91 —	91% —
***Temporal variables***						
**RD**	s	3.8 ± 0.5 (3.5–4.0)	3.7 ± 0.6 (3.3–4.1)	3.9 ± 0.6 (3.5–4.3)	0.50	58%
**RT**	s	8.0 ± 1.0 (7.4–8.5)	7.8 ± 1.2 (7.0–8.7)	8.1 ± 1.0 (7.4–8.8)	0.69	71%
**W:R**		0.49 ± 0.07 (0.45–0.52)	0.49 ± 0.09 (0.42–0.56)	0.49 ± 0.05 (0.45–0.52)	0.88	88%
**NS**	shots·rally^-1^	4.2 ± 0.5 (3.9–4.4)	4.1 ± 0.5 (3.8–4.5)	4.2 ± 0.6 (3.7–4.6)	0.80	81%
**RS**	shots·s^-1^	1.09 ± 0.09 (1.05–1.14)	1.15 ± 0.11 (1.07–1.23)	1.04 ± 0.03* (1.02–1.06)	0.03	56%
***Physiological variables***						
$\dot{V}O_{2\max}$	mL·kg^-1·^min^-1^	45.5 ± 5.3 (42.8–48.2)	42.9 ± 4.2 (40.9–44.9)	48.1 ± 5.8 (45.3–50.8)	0.08	49%
$\dot{V}O_{2\mathrm{MEAN}}$	mL·kg^-1·^min^-1^	29.5 ± 3.8 (27.6–31.5)	30.7 ± 4.7 (28.5–33.0)	28.5 ± 3.0 (27.1–29.9)	0.30	51%
$\dot{V}O_{2\mathrm{PEAK}}$	mL·kg^-1·^min^-1^	41.9 ± 5.3 (39.2–44.6)	42.3 ± 6.6 (39.2–45.5)	41.5 ± 4.8 (39.2–43.8)	0.78	78%
**HR**_**MEAN**_	bpm	142 ± 11 (136–147)	145 ± 11 (139–150)	138 ± 12 (133–144)	0.34	53%
**HR**_**PEAK**_	bpm	166 ± 14 (159–173)	169 ± 12 (163–174)	164 ± 17 (155–172)	0.55	61%
**[La]**_**MEAN**_	mmol.L^-1^	1.4 ± 0.4 (1.2–1.6)	1.6 ± 0.4 (1.4–1.8)	1.2 ± 0.4 (1.0–1.4)	0.16	48%
**[La]**_**PEAK**_	mmol.L^-1^	1.8 ± 0.6 (1.5–2.1)	1.9 ± 0.6 (1.6–2.2)	1.7 ± 0.7 (1.4–2.1)	0.59	64%

W_OXID_: Energetic contribution from oxidative pathway; W_PCr_: Energetic contribution from phosphagen pathway; W_[La]_: Energetic contribution from glycolytic pathway; W_ANAER_: Energetic contribution from anaerobic pathways (sum of phosphagen and glycolytic pathways); W_TOTAL_: Sum of all energy systems contribution; RD: rally duration; RT: rest time between rallies; W:R: work-to-rest ratio; NS: number of shots per rally; RS: rate of shots per rally; $\dot{V}O_{2\max}$: Maximal $\dot{V}O_{2}$ during the GXT; $\dot{V}O_{2\mathrm{MEAN}}$: Mean of $\dot{V}O_{2}$ during the simulated matches; $\dot{V}O_{2\mathrm{PEAK}}$: Highest $\dot{V}O_{2}$ value during the simulated matches; HR_MEAN_: Mean of HR during the simulated matches; HR_PEAK_: Highest HR value during the simulated matches; [La]_MEAN_: Mean of [La] during the simulated matches; [La]_PEAK_: Highest [La] value during the simulated matches. 1-β: Statistical power.

*P < 0.05.

### Comparison between offensive and all-round playing styles

In total, 7 offensive players and 8 all-round players were analyzed. Again, the oxidative systems made major contributions during the simulated matches (above 96% of the total energy amount expended), and there were no significant differences between offensive and all-round players for all energy systems, W_ANAER_ and the W_TOTAL_. Only the rate of shots per rally was significantly different between offensive and all-round playing style (offensive: 1.15 ± 0.11 shots·s^-1^; all-round: 1.04 ± 0.03 shots·s^-1^ –*P* = 0.03). None of physiological variable ($\dot{V}O_{2\mathrm{MEAN}},\;\dot{V}O_{2\mathrm{PEAK}}$, HR_MEAN_, HR_PEAK_, [La]_MEAN_ and [La]_PEAK_) presented significant differences between offensive and all-round players (*P* > 0.16) (Table 1).

**Fig 1:**
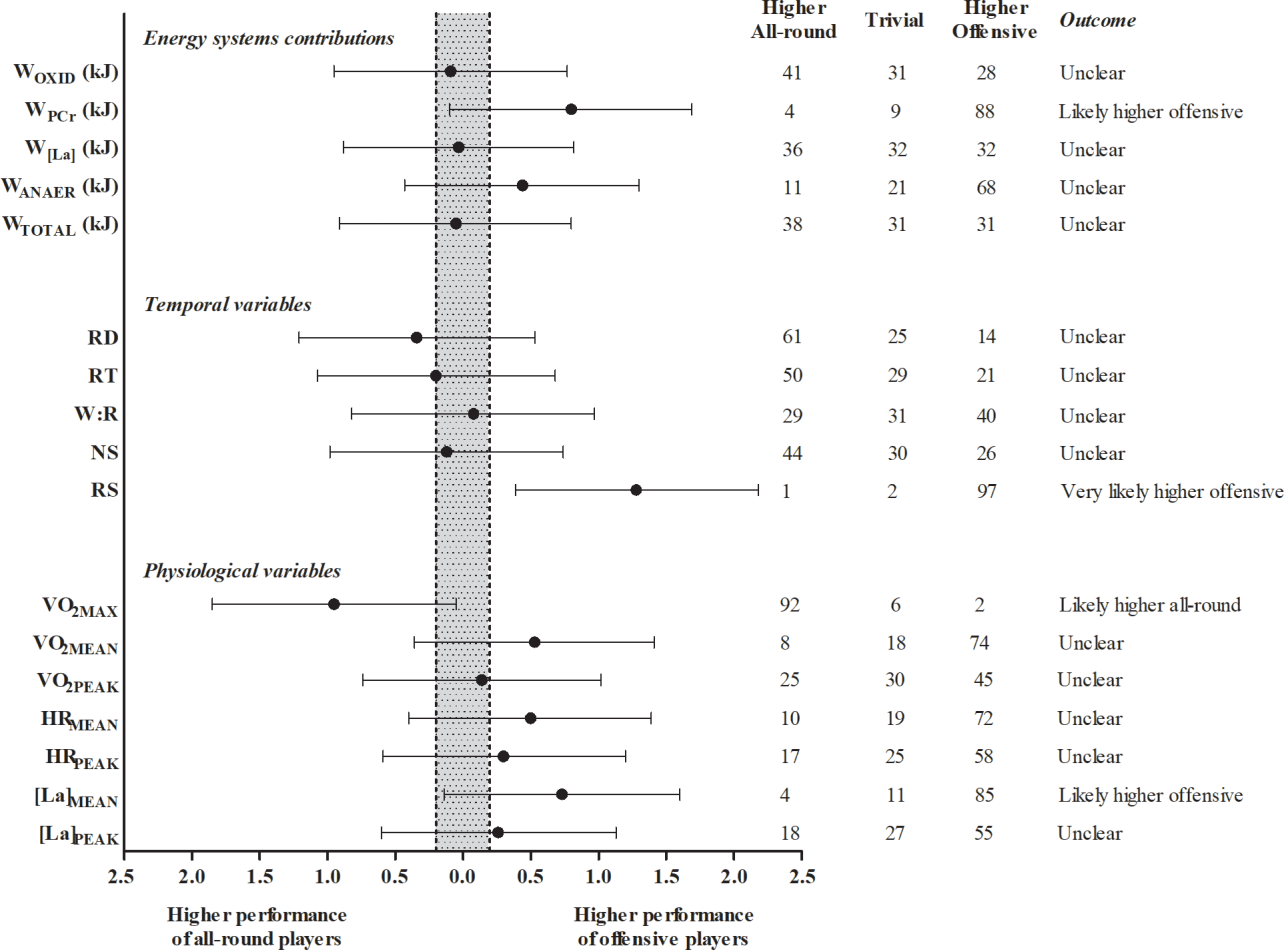
Magnitude-based outcomes for energy systems contributions, temporal variables and physiological variables of all-round and offensive playing styles.

The magnitude-based analysis presented *Very likely lower* rally duration, *Likely lower* W_PCr_, *Likely higher* $\dot{V}O_{2\max}$ and *Likely lower* [La]_MEAN_ for all-round players when compared with offensive players (Fig 1).

Offensive players presented strong associations of number of shots per rally with W_PCr_ (r = -0.86, *P* = 0.01) and W_ANAER_ (r = -0.90, *P* = 0.01); while all-round players presented strong associations of W_OXID_ with rally duration and number of shots per rally (r = 0.76, *P* = 0,03 and r = 0.76, *P* = 0,03, respectively) as well as of W_TOTAL_ with rally duration and number of shots per rally (r = 0.75, *P* = 0.03 and r = 0.75, *P* = 0.03, respectively) (Table 2).

**Table 2:** Pearson’s correlation (r) between energy systems contribution and temporal variables analyzed by playing style.

	RD	RT	W:R	NS	RS
**W**_**OXID**_					
Offensive	0.27	0.14	0.11	0.24	-0.66
All-round	0.76*	0.58	0.28	0.77*	-0.06
**W**_**PCr**_					
Offensive	-0.62	-0.69	0.02	-0.86*	0.71
All-round	0.05	0.58	-0.56	0.12	0.43
**W**_**[La]**_					
Offensive	-0.33	-0.28	-0.05	-0.47	0.13
All-round	0.37	0.04	0.42	0.33	-0.11
**W**_**ANAER**_					
Offensive	-0.64	-0.67	-0.01	-0.90*	0.59
All-round	0.34	0.21	0.19	0.33	0.04
**W**_**TOTAL**_					
Offensive	0.24	0.11	0.11	0.20	-0.64
All-round	0.75*	0.59	0.26	0.75*	-0.06

RD = rally duration; RT = rest time between rallies; W:R = work-to-rest ratio; NS = number of shots per rally; RS = rate of shots per rally; W_OXID_ = Energetic contribution from oxidative pathway; W_PCr_ = Energetic contribution from phosphagen pathway; W_[La]_ = Energetic contribution from glycolytic pathway; W_ANAER_ = Energetic contribution from anaerobic pathways (sum of phosphagen and glycolytic pathways); W_TOTAL_ = Sum of all energy systems contribution.

*P < 0.05.

Regarding the associations of physiological variables and temporal actions, only all-round players showed strong correlations of $\dot{V}O_{2\max}$ with rally duration and number of shots per rally (r = 0.85, *P* = 0.02 and r = 0.81, *P* = 0.03, respectively) (Table 3).

**Table 3:** Pearson’s correlation (r) between physiological variables and temporal variables analyzed by playing style.

	RD	RT	W:R	NS	RS
$\dot{V}O_{2\max}$					
Offensive	-0.21	-0.47	-0.11	-0.55	0.37
All-round	0.85*	0.55	0.71	0.81*	-0.37
$\dot{V}O_{2\mathrm{MEAN}}$					
Offensive	-0.62	-0.16	-0.59	-0.38	0.42
All-round	-0.09	0.02	-0.13	-0.17	-0.46
$\dot{V}O_{2\mathrm{PEAK}}$					
Offensive	-0.59	-0.25	-0.53	-0.45	0.38
All-round	0.37	0.15	0.32	0.33	-0.29
**HR**_**MEAN**_					
Offensive	-0.30	0.21	-0.25	-0.06	0.51
All-round	0.24	0.53	-0.35	0.13	-0.59
**HR**_**PEAK**_					
Offensive	-0.38	0.14	-0.33	-0.16	0.34
All-round	0.56	0.67	0.00	0.49	-0.43
**[La]**_**MEAN**_					
Offensive	-0.63	-0.04	-0.46	-0.28	0.48
All-round	-0.03	-0.11	0.08	-0.06	-0.15
**[La]**_**PEAK**_					
Offensive	-0.52	0.05	-0.49	-0.18	0.39
All-round	-0.09	-0.20	-0.12	-0.06	-0.15

RD = rally duration; RT = rest time between rallies; W:R = work-to-rest ratio; NS = number of shots per rally; RS = rate of shots per rally; $\dot{V}O_{2\max}$: Maximal $\dot{V}O_{2}$ during the GXT; $\dot{V}O_{2\mathrm{MEAN}}$: Mean of $\dot{V}O_{2}$ during the simulated matches; $\dot{V}O_{2\mathrm{PEAK}}$: Highest $\dot{V}O_{2}$ value during the simulated matches; HR_MEAN_: Mean of HR during the simulated matches; HR_PEAK_: Highest HR value during the simulated matches; [La]_MEAN_: Mean of [La] during the simulated matches; [La]_PEAK_: Highest [La] value during the simulated matches.

*P < 0.05.

## Discussion

The main findings of the study were there were no significant differences of energy systems contributions between offensive and all-round players, however the magnitude-based inference showed lower W_PCr_ and higher $\dot{V}O_{2\max}$ values for all-round players when compared with offensive players. Regarding the temporal variables, only the rate of shots per rally presented significantly higher values for offensive players when compared to all-round players. Strong negative correlations were found between W_PCr_ and number of shots per rally for offensive players while strong correlations were found between W_OXID_ and $\dot{V}O_{2\max}$ with rally duration and number of shots per rally for all-round players. Finally, W_OXID_ the main energy system contributor (~97%), followed by the W_PCr_ (~2%), and W_[La]_ systems (~1%).

Previous studies highlighted that the oxidative system has important functions in racket sports [19–22], including the replenishment of phosphocreatine to produce energy in periods of transition of low to high intensity actions and also to allow several repetitions of high-intensity efforts [23,24]. The predominance of the oxidative contribution for both groups could be mainly explained by the low energy demand during the matches [4,25], which is indicated by the quite low values of $\dot{V}O_{2\mathrm{MEAN}}$ (29.5 ± 3.8 mL·kg^-1^·min^-1^; ~66% of $\dot{V}O_{2\max}$), HR_MEAN_ (142 ± 11 bpm) and [La]_MEAN_ (1.4 ± 0.4 mmol·L^-1^). In addition, the long length of rest time between rallies in relation to rally duration and consequently low values of work-to-rest ratio (0.49 ± 0.07) also support the greater contribution of W_OXID_.

Only all-round players presented strong significant correlations of W_OXID_ and $\dot{V}O_{2\max}$ with rally duration and number of shots per rally. These findings are similar to those reported by Zagatto et al. [5] that identified significant correlations of W_OXID_ with rally duration (r = 0.81) and number of shots per rally (r = 0.77) in a general approach on the energetics of table tennis without discrimination between distinct playing styles. Nevertheless, all-round players presented lower rates of shots per rally than offensive players, with all-round players performing a smaller number of strokes per time. These results suggest that all-round players challenge the W_PCr_ energy system to a lesser extent and rely slightly more on the oxidative system.

Lanzoni and co-workers [1] investigated different game features between top-class European (generally assumed as all-round players) and Asian (generally assumed as offensives) table tennis players and confirmed the higher frequency of utilization of aggressive strokes as forehand topspin and forehand top-counter-top by Asian players. In general, all-round players prefer to return the ball and to force opponent´s error, which leads to less aggressive strokes and longer rallies. This strategy requires less actions with maximal effort and thus less reliance on the phosphagen pathway, which may explain the lack of significant correlations of W_PCr_ and temporal variables for all-round players.

However, despite the anaerobic components presented lower contribution compared with the oxidative metabolism (less than ~3.5% for all components), which could be a consequence of the effective playing time of just a quarter of the total match duration, the phosphagen and glycolytic energy pathways still a relevant energetic component during short effort periods. Zagatto et al. [5] reported that the anaerobic capacity estimated via maximal accumulated oxygen deficit is significantly correlated with the rate of shots per rally, that is, how higher the rate of shots per rally, higher will be the anaerobic energy demand. Taking togheter, this results clearly indicates that players with greater anaerobic capacity can play at higher intensity during the game.

Furthermore, besides the absence of statistical significance for the relationship between W_PCr_ and rate of shots per rally of offensive style players, a clear trend can be assumed (i.e., r = 0.71, *P* = 0.08 and 1-β = 97%) and highlights the importance of W_PCr_ development for athletes who wish to play the points with greater intensity, since rate of shots per rally could be a variable to represent intensity during table tennis matches. This result is most likely related to the small sample size of each group and must be assumed as a limitation of the study, despite the difficult to access high level players.

## Conclusion

We can conclude that, despite there are no differences in energy system contributions when comparing offensive and all-round playing style players, the playing styles may require specific energy systems demands for higher table tennis performance. In addition, the phosphagen energetic pathway seems to play an important role for table tennis, mainly for offensive style players, being significantly correlated with indicators of intensity of the match, as well as the oxidative pathway seems to be the most important energetic pathway for all-round players. With the new evidences provided by the present study regarding the energy demand and temporal variables of table tennis, will be possible optimizing training programs and reach better results according specific playing styles.

## Supporting information

S1 Spreadsheet (XLSX)
